# Effects of vitamin D_3_ and calcium supplementation on bone of young adults after thyroidectomy of differentiated thyroid carcinoma

**DOI:** 10.1007/s12020-025-04195-x

**Published:** 2025-03-06

**Authors:** Lei Sun, Xiaoyun Lin, Naishi Li, Qian Zhang, Yan Jiang, Ou Wang, Weibo Xia, Xiaoping Xing, Xiaoyi Li, Mei Li

**Affiliations:** 1 Department of Endocrinology, Key Laboratory of Endocrinology of National Ministry of Health, Peking Union Medical College Hospital, Chinese Academy of Medical Science and Peking Union Medical College, Beijing, 100730 China; 2 Department of General Surgery, Peking Union Medical College Hospital, Chinese Academy of Medical Science and Peking Union Medical College, Beijing, 100730 China

**Keywords:** Differentiated thyroid cancer, TSH suppression therapy, Vitamin D, Calcium, Bone mineral density

## Abstract

**Purpose:**

Differentiated thyroid carcinoma (DTC) is the most frequent endocrine cancer, with a high incidence in young population. Patients with postoperative DTC are usually considered with increased risk of bone loss, possibly due to the thyroid stimulating hormone (TSH) suppression therapy. However, it remains unclear whether vitamin D and calcium supplementation is beneficial on bone metabolism of young patients with vitamin D malnutrition after thyroidectomy of DTC.

**Methods:**

In this one-year prospective study, adult men younger than 50 years or premenopausal women with DTC and vitamin D insufficiency or deficiency were enrolled after thyroidectomy, who were administered with daily supplements of 1000–2000 IU vitamin D_3_ and 600 mg of elemental calcium (calcium-D_3_) or not. Propensity score matching (PSM) was applied to identify baseline-matched cohorts.

**Results:**

A total of 458 patients with a median age of 37 (range 21–50) years were enrolled, with 94 (20.5%) patients supplemented with calcium-D_3_. After PSM, we identified baseline-matched cohorts of 246 DTC patients, of which 82 patients were supplemented with calcium-D_3_ and 164 were not. After 12 months’ supplementation, lower serum levels of β-CTX (0.27 ± 0.15 vs. 0.35 ± 0.18 ng/ml, *P* = 0.004), PTH (36.2 ± 12.7 vs. 45.2 ± 14.6 pg/ml, *P* < 0.001) and higher BMD at lumbar spine (1.8% vs. 0.7%, *P* = 0.050) and total hip (1.1% vs. −0.4%, *P* < 0.001) were observed compared to the control group. Among all the 458 patients, increase of 25OHD levels was closely associated with decrease of PTH, ALP and β-CTX levels and improvement in total hip BMD throughout the one-year study period.

**Conclusion:**

Vitamin D and calcium supplements can reduce PTH levels and bone loss, possibly contributing to protecting bone of young DTC patients with vitamin D malnutrition after thyroidectomy.

## Introduction

The incidence of differentiated thyroid carcinoma (DTC) is significantly increased for the last 30 years, and it is now the most frequent endocrine cancer [1]. After initial surgical treatment of DTC, thyroid stimulating hormone (TSH) suppressive therapy using exogenous levothyroxine is recommended to inhibit the growth of residual neoplastic tissue and improve long-term survival of DTC patients [2]. As thyroid hormone can regulate the initiation and duration of the bone remodeling cycle in the human skeleton, it has been reported that exogenous subclinical thyrotoxicosis might accelerate bone turnover and increase the risks of fracture in postmenopausal women [3–6]. DTCs are quite common in young adults, but the effects of TSH suppression therapy on bone of premenopausal women and young men with DTCs remain controversial [7, 8].

Moreover, high prevalence of vitamin D deficiency has been reported worldwide, and approximately 80% of Chinese general population are suffering from vitamin D insufficiency or deficiency (25OHD levels less than 30 ng/ml) [9, 10]. Vitamin D plays an important role in homeostasis regulation of calcium and phosphorus, and vitamin D malnutrition will increase the risk of skeletal and muscular diseases, including osteoporosis, osteomalacia, bone fracture, deceased muscle strength, and fall [11]. Severe vitamin D deficiency can increase bone resorption and result in secondary hyperparathyroidism, thus further accelerating bone loss [11]. It is reasonable to speculate that the risk of osteoporosis may be increased in premenopausal women and young men during co-occurrence of subclinical hyperthyroidism and poor vitamin D status. However, the impact of poor vitamin D status and TSH suppressive therapy on bone metabolism of young patients with DTC has not been elucidated comprehensively. Moreover, postulated protective effect of vitamin D and calcium supplements on bone has not yet been validated in young patients with DTC and vitamin D malnutrition, although vitamin D and calcium have long been regarded as fundamental treatment for primary osteoporosis.

Therefore, the present study aimed to prospectively assess the effects of supplementation of vitamin D_3_ and calcium on bone turnover biomarkers and bone mineral density (BMD) of premenopausal women and young men after thyroidectomy of DTC.

## Patients and methods

### Study design and participants

This was a one-year single-center non-randomized concurrent controlled trial (Non-R). Participants were recruited from Endocrinology department and General Surgery department of Peking Union Medical College Hospital (PUMCH) from January 2019 to January 2022.

Premenopausal women (age greater than or equal to 20 years) and young men (aged 20–50 years) with pathological diagnosis of DTC were eligible for inclusion at least 3 months after thyroidectomy. We excluded patients who had taken other agents to affect bone metabolism, such as bisphosphonates, denosumab, teriparatide, calcitonin, glucocorticoid, and so on. Patients with postoperative hypoparathyroidism, primary hyperparathyroidism, distant metastasis of DTC were also discarded from the analysis. Patients with diagnosis of osteoporosis who were suggested to initiate anti-osteoporotic therapy and patients with vitamin D sufficiency were also discarded from the study.

The participants were divided into vitamin D_3_ plus calcium (calcium-D_3_) supplementation group versus control group based on patients’ wishes and doctors’ advice. Patients in the calcium-D_3_ group were administered with supplementation of 1000IU or 2000 IU cholecalciferol plus 600 mg elemental calcium daily according to baseline 25OHD levels, while patients in control group did not receive any supplements with vitamin D_3_ and calcium.

The study was approved by the Scientific Ethnics Committee of PUMCH. Written informed consents were obtained from all participants at the time of enrollment.

### Biochemical index assessment

Fasting venous blood samples were collected from the participants. Serum concentrations of TSH, free triiodothyronine (FT_3_) and free thyroxine (FT_4_) were detected by chemiluminescence immunoassay (Atellica, Siemens Healthineers, USA). Serum levels of calcium (Ca), phosphate (P), and total alkaline phosphatase (ALP, a bone formation marker) were detected by an automatic biochemical analyzer (ADVIA 1800, Siemens Healthineers, USA). Serum levels of 25-hydroxyvitamin D (25OHD), intact parathyroid hormone (PTH), and β-isomerized carboxy-telopeptide of type I collagen (β-CTX, a bone resorption marker) were measured using an automatic analyzer for electrochemiluminescence (Cobas, Roche Diagnostics, Switzerland). Blood biochemistry of the patients was measured at baseline, 6 and 12 months during the follow-up in the clinical laboratory of PUMCH.

Subclinical hyperthyroidism was defined as serum TSH levels less than 0.5 mIU/L with normal serum concentrations of FT_3_ (1.80–4.10 pg/ml) and FT_4_ (0.81–11.89 ng/dl) [12]. Severe deficiency, deficiency, insufficiency and sufficiency of vitamin D were defined as serum 25OHD levels less than 10 ng/ml, 20 ng/ml, 30 ng/ml, and higher or equal to 30 ng/ml, respectively [13].

### Bone mineral density measurement

Body height and weight were measured using a Harpenden stadiometer (Seritex Inc., East Rutherford, USA). Areal BMD at lumbar spine (LS, L_2_–L_4_), femoral neck, trochanter and total hip (TH) were measured by dual energy X-ray absorptiometry (DXA, Lunar Prodigy Advance, GE Healthcare, USA). Phantom testing was conducted daily by the DXA device for calibration and quality check to guarantee the accuracy of BMD measurement. The coefficients of variation (CVs) of DXA measurements were 1.1% −1.7%. Values of areal BMD were converted to age- and gender-specific Z-scores using data of Asian general population. Participants receiving calcium-D_3_ supplements completed BMD assessment at baseline, 6 months and 12 months of treatment, and BMD assessment of control group was conducted at baseline and 12 months of follow up.

### Statistical analysis

Kolmogo-rov-Smirnov test was performed to test normal distribution of quantitative data. Normally distributed data (including BMD and serum levels of ALP, β-CTX, 25OHD and PTH) were expressed as mean ± standard deviation (SD). Student’s t-test was used to compare these normally distributed continuous variables. Data of abnormal distribution (including serum levels of TSH, duration after thyroidectomy) were expressed as median and interquartile range and the comparison between vitamin D group versus control group was completed with Mann-Whitney U test. Pearson’s chi-square test or Fisher’s exact test was used to compare qualitative data. The bivariate Pearson correlation coefficients between the change of 25OHD levels and other parameters throughout the study were calculated.

Given the differences of baseline variables between the two groups, propensity score matching was applied to identify a cohort of participants with similar characteristics, and thus clinical outcomes during the follow-up were compared between matched groups. The propensity score was estimated from a logistic model to form a sample consisting of participants taking calcium-D_3_ or not by 1:2 using the optimal matching algorithm. Clinical variables including age, gender, serum levels of 25OHD, PTH and β-CTX, BMD at lumbar spine and total hip were incorporated in the analysis.

Two-tailed *P* value less than 0.05 was considered as statistical significance. The statistical analyses were performed using SPSS software version 25.0 (SPSS Inc., USA) and R version 4.1.3 (R Foundation for Statistical Computing, Austria). Graphs were drawn using GraphPad Prism software version 8.0 (GraphPad, USA).

## Results

### Baseline characteristics

**Fig. 1:**
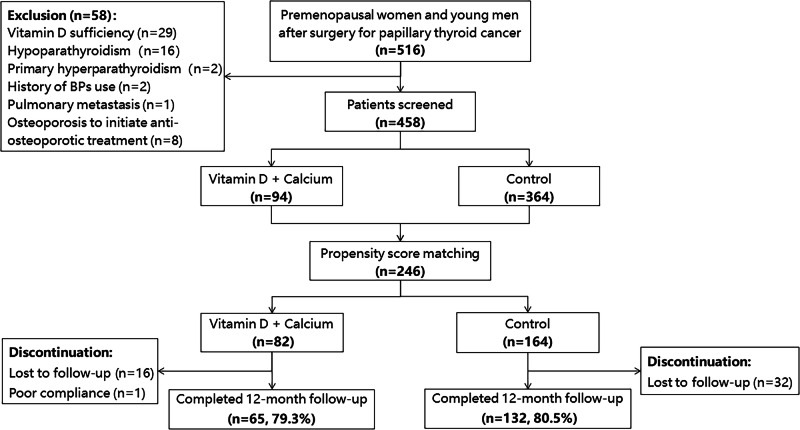
Flowchart of the study

A total of 458 patients (138 men and 320 women) with a median age of 37 (range 21–50) years were enrolled in this study (Fig. 1). 94 (20.5%) patients were administered with calcium-D_3_, with the remaining 364 (79.5%) receiving no supplements of calcium-D_3_. Patients’ baseline characteristics were presented in Table 1. In the unadjusted cohorts, patients administered with calcium-D_3_ presented with lower 25OHD (13.7 ± 3.8 vs. 16.8 ± 5.4 ng/ml; *P* < 0.001) and higher PTH (46.7 ± 18.6 vs. 41.4 ± 15.5 pg/ml; *P* = 0.015) levels, and lower BMD at both lumbar spine (1.22 ± 0.12 vs. 1.27 ± 0.14 g/cm^2^; *P* = 0.002) and total hip (0.99 ± 0.12 vs. 1.02 ± 0.12 g/cm^2^; *P* = 0.017). Age, BMI, serum concentrations of TSH, ALP and β-CTX were similar between patients in two groups.

**Table 1:** Baseline characteristics of study participants

	Before propensity score matching			After propensity score matching		
	Vitamin D group *n* = 94	Control group *n* = 364	*P* value	Vitamin D group *n* = 82	Control group *n* = 164	*P* value
Age (yr)	35.3 ± 5.8	36.1 ± 5.5	0.223	35.2 ± 5.9	35.5 ± 5.8	0.717
Sex, Male (%)	22 (23.4)	116 (31.9)	0.130	19 (23.2)	39 (23.8)	0.915
BMI (kg/m^2^)	23.4 ± 3.7	24.0 ± 3.7	0.192	23.6 ± 3.7	23.5 ± 3.7	0.836
Total thyroidectomy (%)	41 (43.6)	168 (46.2)	0.728	35 (42.7)	70 (42.7)	1.000
Time to operation (yr)	0.62 (0.22, 2.35)	1.02 (0.15, 2.64)	0.290	0.55 (0.22, 2.35)	1.04 (0.15, 2.6)	0.610
TSH (mIU/L)	0.62 (0.15, 1.57)	0.66 (0.18, 1.59)	0.947	0.62 (0.15, 1.57)	0.68 (0.17, 1.66)	0.916
<0.1	17 (18.1)	68 (18.7)	0.973	13 (15.9)	32 (19.5)	0.395
0.5	23 (24.5)	85 (23.4)		22 (26.8)	32 (19.5)	
>0.5	54 (57.4)	210 (57.9)		47 (57.3)	100 (61.0)	
25OHD (ng/ml)	13.7 ± 3.8	16.8 ± 5.4	**<0.001**	13.8 ± 3.9	14.3 ± 4.5	0.148
<10	14 (14.9)	37 (10.2)	**<0.001**	11 (13.4)	28 (17.1)	0.347
10 ~ 20	73 (77.7)	225 (61.8)		64 (78.0)	114 (69.5)	
20 ~ 30	7 (7.4)	102 (28.0)		7 (8.5)	22 (13.4)	
PTH (pg/ml)	46.7 ± 18.6	41.5 ± 15.5	**0.007**	45.5 ± 18.1	45.3 ± 17.1	0.223
≤65	77 (85.6)	335 (93.6)	**0.017**	72 (87.8)	148 (90.2)	0.557
>65	13 (14.4)	23 (6.4)		10 (12.2)	16 (9.8)	
ALP (U/L)	68 ± 20	67 ± 17	0.748	67 ± 17	68 ± 20	0.794
β-CTX (ng/ml)	0.37 ± 0.19	0.36 ± 0.17	0.554	0.37 ± 0.19	0.38 ± 0.17	0.769
LS BMD (g/cm^2^)	1.223 ± 0.119	1.273 ± 0.136	**0.002**	1.224 ± 0.119	1.232 ± 0.118	0.633
Z-score	0.25 ± 1.09	0.59 ± 1.04	**0.009**	0.24 ± 1.10	0.31 ± 0.92	0.640
FN BMD (g/cm^2^)	0.945 ± 0.110	0.979 ± 0.120	**0.018**	0.945 ± 0.112	0.955 ± 0.109	0.505
Z-score	0.03 ± 0.86	0.31 ± 0.88	**0.012**	0.03 ± 0.87	0.15 ± 0.80	0.290
TROCH BMD (g/cm^2^)	0.747 ± 0.110	0.787 ± 0.108	**0.003**	0.748 ± 0.111	0.762 ± 0.097	0.295
Z-score	−0.38 ± 0.84	−0.09 ± 0.86	**0.007**	−0.38 ± 0.84	−0.25 ± 0.77	0.251
TH BMD (g/cm^2^)	0.988 ± 0.118	1.023 ± 0.117	**0.015**	0.990 ± 0.119	0.997 ± 0.105	0.630
Z-score	0.07 ± 0.88	0.36 ± 0.83	**0.006**	0.08 ± 0.89	0.18 ± 0.74	0.332

Values were given as mean ± SD, number (proportion) or median (interquartile range). Bold *P* values indicated significant differences between groups

*BMI* Body mass index; *TSH* Thyroid stimulating hormone; *25OHD* 25-Hydroxyvitamin D; *PTH* Parathyroid hormone; *ALP* Alkaline phosphatase, *β-CTX* β-Isomerized carboxy-telopeptide of type I collagen; *BMD* Bone mineral density; *LS* Lumbar spine; *FN* Femoral neck; *TROCH* Trochanter; *TH* Total hip

After performing propensity core matching, there were a total of 246 patients composed of 188 (76.4%) women and 58 (23.6%) men and the median age was 35 (range 21–50) years. Two groups (82 with calcium-D_3_ and 164 controls) were well matched, with no significant differences in baseline characteristics (Table 1). Concentrations of 25OHD (13.8 ± 3.9 vs. 14.3 ± 4.5 ng/ml, *P* = 0.148) and PTH (45.5 ± 18.1 vs. 45.3 ± 17.1 pg/ml, *P* = 0.223) and BMD at all sites were comparable at baseline between the two groups.

### Changes of blood biochemical indexes

**Fig. 2:**
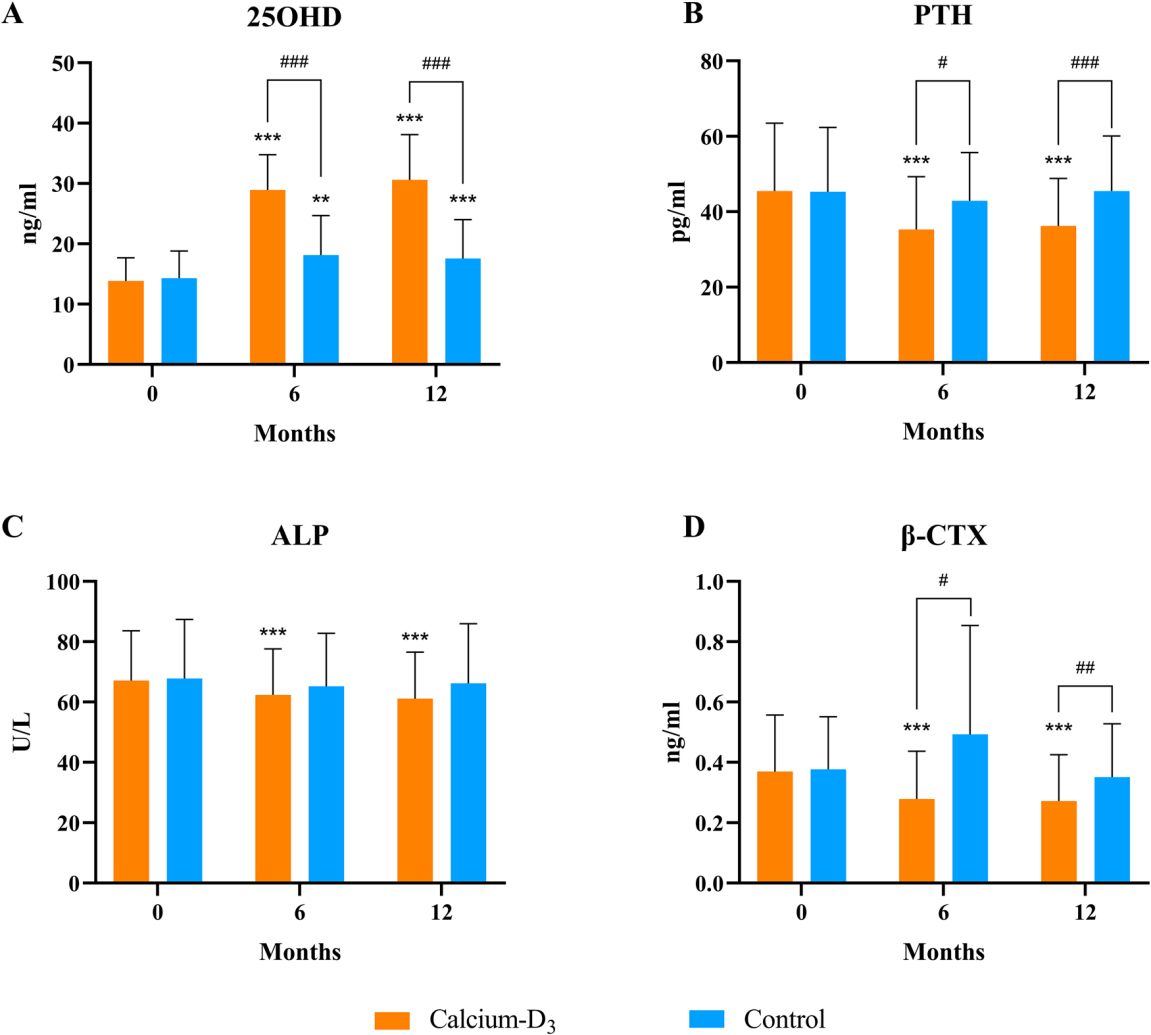
Serum levels of 25OHD, PTH and bone turnover markers throughout the study period. **A** Serum levels of 25OHD during the follow-up. **B** Serum levels of PTH during the follow-up. **C** Serum levels of ALP during the follow-up. **D** Serum levels of β-CTX during the follow-up. 25OHD: 25-hydroxyvitamin D; PTH parathyroid hormone, ALP alkaline phosphatase, β-CTX β-isomerized carboxy-telopeptide of type I collagen. Data were shown as mean and standard error. *: *P* < 0.05, **: *P* < 0.01, ***: *P* < 0.001 indicated significant difference compared with baseline. #: *P* < 0.05, ##: *P* < 0.01, ###: *P* < 0.001 indicated significant difference between treatment and control groups

A total of 197 (80.1%) of the 246 propensity score-matched patients completed the one-year follow-up, and Fig. 2 summarized the changes of serum bone biochemical indexes during the study period. After supplementation of calcium-D_3_, significant increase of 25OHD levels and decrease of concentrations of PTH, β-CTX and ALP were noted at 6 and 12 months compared with baseline. Serum 25OHD concentrations were significantly higher in patients receiving supplementation of calcium-D_3_ than those in control group (28.9 ± 5.8 vs. 18.1 ± 6.5 ng/ml, *P* < 0.001 at 6 months; 30.6 ± 7.5 vs. 17.6 ± 6.5 ng/ml, *P* < 0.001 at 12 months). Serum levels of PTH in calcium-D_3_ group were significantly lower than control group (35.3 ± 14.0 vs. 42.9 ± 12.9 pg/ml, *P* = 0.013 at 6 months; 36.2 ± 12.7 vs. 45.2 ± 14.6 pg/ml, *P* < 0.001 at 12 months). Accordingly, serum β-CTX levels of patients with calcium-D_3_ supplements were lower than those without supplements (0.28 ± 0.16 vs. 0.49 ± 0.36 ng/ml, *P* = 0.034 at 6 months; 0.17 ± 0.15 vs. 0.35 ± 0.18 ng/ml, *P* = 0.004 at 12 months). However, similar ALP levels between the two groups were observed throughout the study period (62 ± 15 vs. 65 ± 18 U/L, *P* = 0.344 at 6 months; 61 ± 15 vs. 66 ± 20 U/L, *P* = 0.082 at 12 months). Throughout the treatment duration, none of the participants in the calcium-D_3_ group developed hypercalcemia.

### Changes of bone mineral density

**Fig. 3:**
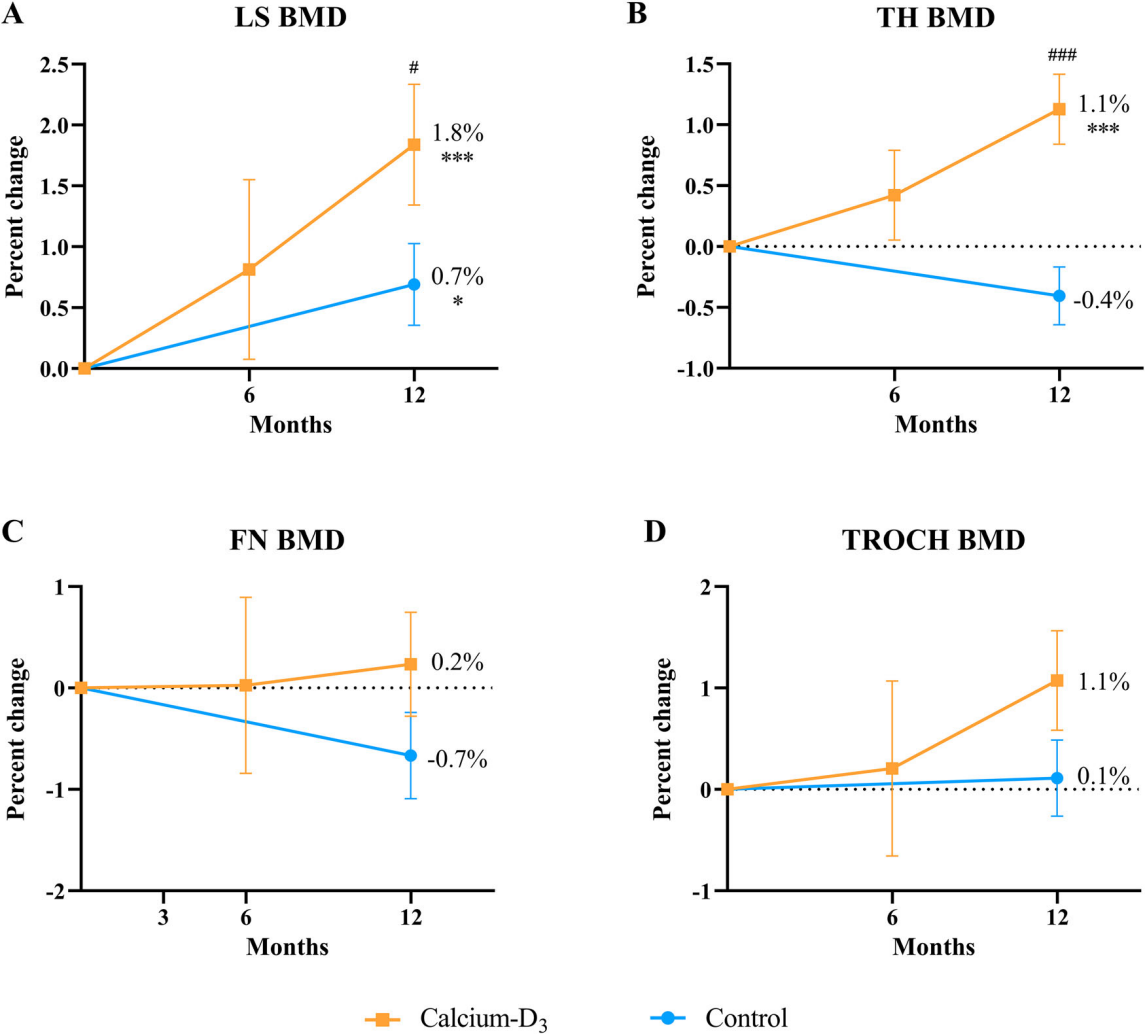
Percentage change of BMD throughout the study period. **A** Percentage change of lumbar spine BMD during the follow-up. **B** Percentage change of total hip BMD during the follow-up. **C** Percentage change of femoral neck BMD during the follow-up. **D** Percentage change of trochanter BMD during the follow-up. BMD bone mineral density, LS lumbar spine, FN femoral neck, TROCH trochanter, TH total hip. Data were shown as mean and standard error. *: *P* < 0.05, **: *P* < 0.01, ***: *P* < 0.001 indicated significant difference compared with baseline. #: *P* < 0.05, ##: *P* < 0.01, ###: *P* < 0.001 indicated significant difference between treatment and control groups

After one-year supplementation of calcium-D_3_, BMD at the lumbar spine and total hip increased significantly from baseline (LS: 1.216 ± 0.124 g/cm^2^ at baseline vs. 1.238 ± 0.126 g/cm^2^ at 12 months, *P* < 0.001; TH: 0.982 ± 0.114 g/cm^2^ at baseline vs. 0.993 ± 0.113 g/cm^2^ at 12 months, *P* < 0.001; Fig. 3). Mean percentage changes at 12 months from baseline in BMD at the lumbar spine and total hip were greater in patients with calcium-D_3_ supplements than those in the control group (LS: 1.8 ± 3.4% vs. 0.8 ± 3.1%, *P* = 0.050; TH: 1.1 ± 1.9% vs. −0.4 ± 2.1%, *P* < 0.001). Changes of BMD at femoral neck and trochanter had no significant differences between the two groups.

### Stratified analysis based on TSH level

**Fig. 4:**
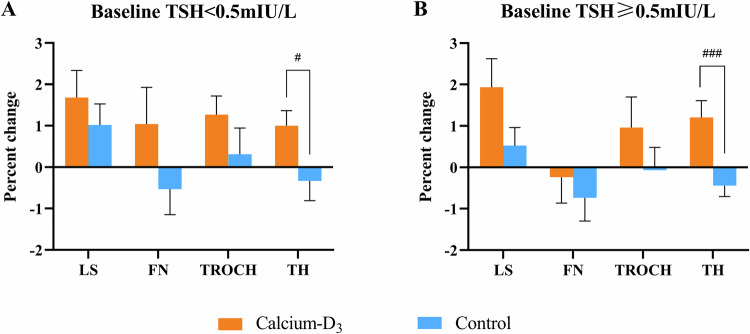
Change of BMD from baseline by treatment group and baseline TSH level. **A** Percentage change of BMD in patients with baseline TSH less than 0.5mIU/L. **B** Percentage change of BMD in patients with baseline TSH higher than or equal to 0.5mIU/L. BMD bone mineral density, LS lumbar spine, FN femoral neck, TROCH trochanter, TH total hip. Data were shown as mean and standard error. *P* < 0.05 indicated significant difference between treatment and control groups

There were 35 of 82 (42.7%) patients with calcium-D_3_ supplements and 64 of 164 (39.0%) patients in control group categorized as subclinical hyperthyroidism (TSH < 0.5mIU/L). In subgroup analysis, significant differences were observed in concentrations of PTH and β-CTX at 12 months between calcium-D_3_ and control groups in patients with or without subclinical hyperthyroidism. There was a greater change of TH BMD in patients with calcium-D_3_ supplements than controls irrespective of baseline TSH status (TSH < 0.5mIU/L: 1.0 ± 1.5% vs. −0.3 ± 2.4%, *P* = 0.050; TSH ≥ 0.5mIU/L: 1.2 ± 2.2% vs. −0.4 ± 1.9%, *P* < 0.001; Fig. 4). Supplemental Table 1 depicted the characteristics and biochemistry changes of the participants by baseline TSH levels.

### Outcomes among the unadjusted cohorts

**Fig. 5:**
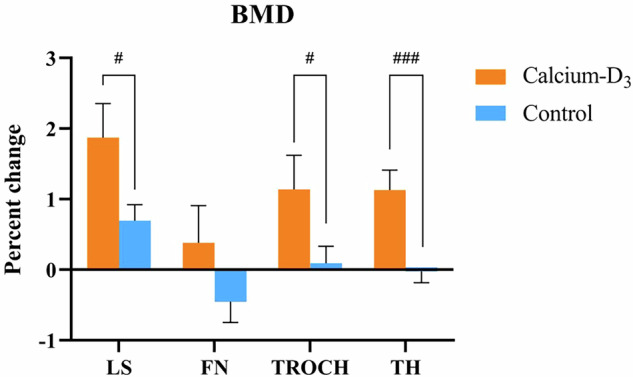
Percentage change of BMD from baseline among unadjusted cohorts. BMD bone mineral density, LS lumbar spine, FN femoral neck, TROCH trochanter, TH total hip. Data were shown as mean and standard error. #: *P* < 0.05, ##: *P* < 0.01, ###: *P* < 0.001 indicated significant difference between treatment and control groups

A comparison of outcomes between the two groups prior to matching (94 treatment vs. 364 control) showed consistent findings: higher 25OHD level, lower PTH and β-CTX levels and greater percentage changes from baseline in BMD at lumbar spine, trochanter and total hip in patients with calcium-D_3_ supplements than controls at 12 months. Between-group differences in BMD at lumbar spine and total hip at baseline became statistically insignificant at 12 months, granting that calcium-D_3_ supplementation helped narrow the BMD gaps at baseline. These data were available in Fig. 5 and Supplemental Table 2.

The changes in serum 25OHD level throughout the study was negatively associated with changes in PTH, ALP and β-CTX levels in all patients prior to matching (ΔPTH: Pearson r = −0.303, *P* < 0.001; ΔALP: Pearson r = −0.297, *P* < 0.001; Δβ-CTX: Pearson r = −0.266, *P* < 0.001; Table 2). Additionally, improvement of 25OHD status showed a significant correlation with increase in total hip BMD (Pearson r = 0.166, *P* = 0.017).

**Table 2:** Correlation between Δ25OHD and changes of bone metabolic indexes and BMD throughout the study in all patients prior to matching

Variables	Δ25OHD (ng/ml) Pearson coefficient r	*P* value
ΔPTH (pg/ml)	−0.303	**<0.001**
ΔALP (U/L)	−0.297	**<0.001**
Δβ-CTX (ng/ml)	−0.266	**<0.001**
ΔLS BMD (g/cm^2^)	0.094	0.182
ΔFN BMD (g/cm^2^)	0.067	0.339
ΔTROCH BMD (g/cm^2^)	0.085	0.223
ΔTH BMD (g/cm^2^)	0.166	**0.017**

Bold *P* values indicated significance

*Δ* values at 12 months – values at baseline; *25OHD* 25-hydroxyvitamin D; *PTH* parathyroid hormone; *ALP* alkaline phosphatase, *β-CTX* β-isomerized carboxy-telopeptide of type I collagen; *BMD* bone mineral density; *LS* lumbar spine; *FN* femoral neck; *TROCH* trochanter; *TH* total hip

## Discussion

To our knowledge, we prospectively evaluate the effects of calcium-D_3_ supplementation on bone metabolism and BMD for the first time in young adults after thyroidectomy of DTC. We found that such supplements with calcium-D_3_ possibly had bone protection effects through reducing PTH secretion and bone loss compared with control group. Evaluation of outcomes of the unadjusted cohorts showed that increase in serum 25OHD levels was closely associated with decrease in PTH, ALP and β-CTX levels and increase in total hip BMD.

DTC has become one of the most common cancers in young adults, associated with a high survival rate [14]. These patients may be confronted with relatively long-term of TSH suppression therapies to prevent tumor re-growth after the primary surgical treatment. A series of studies revealed that subclinical hyperthyroidism was significantly associated with decreased BMD and increased fracture risk [15–17], and deterioration of bone microarchitecture was observed in postmenopausal women after long-term TSH suppression therapy [18–20]. It is believed that excess thyroid hormone can increase the number and activity of osteoclasts, inhibit the replication of osteoblasts, thus accelerating bone loss and reducing BMD [21]. Moreover, low TSH level might impair bone health by playing an independent role of increasing osteoclastogenesis and decreasing osteoblastogenesis [22]. Although overt hyperthyroidism is expected to be harmful to bone health, the effects of TSH suppression therapy on bone seemed to be related to age [23]. According to previous meta-analyses, TSH suppression therapy decreased BMD in postmenopausal women but not premenopausal women [7, 8, 24]. In the circumstance, the protection of skeleton of young DTC patients receiving TSH suppression therapy is often overlooked.

In this study, we focused on young DTC patients with vitamin D insufficiency or deficiency, which was considered another risk factor of osteoporosis apart from the possible TSH suppressive status [25]. Vitamin D malnutrition was associated with secondary hyperparathyroidism and bone loss in postmenopausal women [26]. Previous studies have demonstrated that a combination of vitamin D and calcium had a beneficial role by increasing BMD and muscle strength and reducing fracture risks in elderly population with vitamin D deficiency [27–31]. However, such skeletal protective effects of vitamin D and calcium were ambiguity in general young adults, and empiric supplementation among general population is not recommended [32, 33]. A double-blinded, randomized, placebo-controlled study confirmed that supplementation with vitamin D and calcium for one year played beneficial roles on increasing BMD in underprivileged Bangladeshi premenopausal women [34]. In addition, 6-month daily serving of multi-micronutrients including calcium (600 mg), vitamin D (400IU), and vitamin K (55 mcg) helped decrease serum CTX levels by about 30% among premenopausal women [35]. Nevertheless, a recent meta-analysis revealed that vitamin D supplementation failed to improve BMD at total hip or lumbar spine in healthy premenopausal women [36]. Likewise, in middle-aged healthy men, vitamin D treatment seemed have no significant effects on bone turnover markers, BMD and trabecular bone score [37]. In VITAL study, vitamin D_3_ supplementation did not result in a lower fracture risk among general midlife and older adults who were not selected for vitamin D deficiency, low bone mass or osteoporosis [38]. Overall, the inconsistent outcomes may be related to varying population characteristics at baseline, including age, vitamin D nutritional status, bone mineral density, and so on. In addition, difference in dosage or duration of supplementation of vitamin D might play a role.

Prospective studies focusing on bone protection in patients undergoing TSH suppression therapy, particularly among young adults, remain scarce. Kung et al. firstly found that supplement of 1000 mg calcium daily for two years could prevent bone loss in postmenopausal women who received TSH suppression therapy [39]. In this study, we observed that supplementation of vitamin D_3_ and calcium not only could reduce serum levels of PTH and β-CTX, but also could slightly increase BMD of young patients with DTC and vitamin D insufficiency or deficiency. To the best of our knowledge, this was the first longitudinal study to determine the effects of calcium and vitamin D_3_ supplementation on bone metabolism of young DTC patients undergoing TSH suppressive therapy. Heretofore, another small cross-sectional study revealed that patients receiving daily doses of 500–2000 mg calcium and 400–1600IU vitamin D_3_ had significantly higher BMD than patients without supplements after long-term TSH suppressive therapy since DTC surgery at a young age [40], which was in accordance with the results of this study. However, as both studies had a relatively small sample size, the bone protective effect of vitamin D needs to be further confirmed in large long-term studies. Intriguingly, stratified analysis based on TSH level of this study revealed that even in patients with normal levels of TSH, the beneficial effects of vitamin D_3_ supplements on bone still existed. It is speculated that DTC itself might be a potential risk factor for osteoporosis [41], that is, Vitamin D malnutrition and some undetermined factors other than suppressive TSH status may also lead to bone deterioration, which appeared to be partly rescued by vitamin D_3_ and calcium supplementation.

As an important steroid hormone involved in the calcium and phosphorus metabolism, vitamin D helps maintain a positive calcium balance, reduce bone loss, regulate PTH secretion [42]. Dan et al. demonstrated that bone resorptive markers increased significantly in the mice with thyrotoxicosis, and supplementation with vitamin D reduced the number of osteoclasts and improved BMD and trabecular bone architecture of these mice [43]. The mice with thyrotoxicosis presented with decreased expression of β-catenin and increased expression of receptor activator of NF-κB ligand (RANKL) resulting in increased activity of osteoclasts [43]. Supplementation with vitamin D can increase the expression of β-catenin and inhibit the expression of RANKL, thus improving bone formation and reducing bone loss [44, 45].

We found that DTC patients receiving vitamin D_3_ and calcium supplementation exhibited improvement in BMD at lumbar spine and total hip. However, patients in control group did not present apparent bone loss during the one-year follow-up, which indicated that vitamin D malnutrition together with TSH suppression therapy for one year did not cause a significant decrease in BMD of premenopausal women and young men. Another Chinese prospective study also suggested that one-year TSH suppression therapy did not show detrimental effects on bone turnover markers and BMD in DTC patients [46]. We suppose that the changes in BMD and bone microstructure among such patients remain to be investigated in larger cohorts with longer follow-up to figure out whether Vitamin D malnutrition and TSH suppression can collaboratively undermine bone health among young DTC patients.

Overall, this study showed that supplements with vitamin D and calcium could reduce PTH levels and bone loss, possibly contributing to protecting bone of young DTC patients after thyroidectomy, which had clinical values in reminding doctors and patients to evaluate the nutritional status of vitamin D and to provide calcium and vitamin D supplements for the patients after the surgery. However, long-term supplementation with calcium and vitamin D may increase the patient’s medical expenses, increase the risk of kidney stones, and may even have little effect on reducing future risk of osteoporosis. Therefore, we still require to weigh the pros and cons when deciding whether young DTC patients with TSH suppression therapy after thyroidectomy need long-term supplementation of calcium and vitamin D, especially for patients with sufficient vitamin D.

This study had several strengths. First of all, although osteoporosis and fractures are relatively rare in premenopausal women and men younger than 50 years, optimization of BMD during this period is of great significance for preventing osteoporosis and reducing the risk of fractures [47, 48]. An important difference of this study was the patients included, premenopausal women and men younger than 50 years after thyroidectomy, providing important clinical data about effects of calcium-D_3_ supplementation in these patients whose bone health was conventionally overlooked. Secondly, despite the non-randomized nature of this study, we performed propensity score matching to obtain groups with well-matched baseline characteristics. Thirdly, we conducted a correlation analysis between change of 25OHD level and changes in bone turnover markers and BMD among patients who completed 12-month follow-up, which indirectly verified our results in the adjusted cohorts.

However, several limitations of this study should be clarified. To begin with, this was a study with a relatively small sample size and short follow-up, so it was difficult to observe the outcomes of osteoporotic fracture and incidence of osteoporosis. Also, other risk factors for osteoporosis were not assessed in this study, including smoking, alcohol abuse, physical inactivity, and so forth. Furthermore, our study was not a randomized clinical study. Although propensity score matching is well accepted to balance between-group differences at baseline in observational research, it cannot account for some unmeasured confounding factors [49]. Additionally, serum 25OHD concentrations were closely associated with the season, but we cannot eliminate the potential bias as the participants were recruited over the whole year. What’s more, as most of our participants were virtually further categorized as vitamin D deficiency, the exact threshold of 25OHD concentration for the beneficial effects vitamin D supplementation on bone health could not be determined in this study, which deserves further research [28].

In conclusion, beneficial effects of vitamin D_3_ and calcium supplementation on reducing PTH secretion and increasing BMD are indicated in premenopausal women and young men with vitamin D malnutrition after thyroidectomy, the magnitude of which appears to be associated with the change scope of 25OHD levels. Given the high prevalence of vitamin D deficiency, the current results may have important implications for bone protection among patients with thyroidectomy of DTC. These findings require further investigation in randomized trials with larger population and longer follow-up.

## Supplementary information

Supplemental Tables

## Data Availability

The data that support the findings of this study are available from the corresponding author upon reasonable request.
