# Supplementary material for: Effects of vitamin D3 and calcium supplementation on bone of young adults after thyroidectomy of differentiated thyroid carcinoma
Source: Endocrine. 2025 Mar 6;88(3):836–46. doi: 10.1007/s12020-025-04195-x (PMC12143988; doi:10.1007/s12020-025-04195-x)
Supplement: Supplementary file 1 — Supplemental Tables [file 12020_2025_4195_MOESM1_ESM.docx]

**Supplemental Table 1** Characteristics and outcomes of patients stratified by baseline TSH levels

| **TSH<0.5 mIU/L** | Vit D group  n=35 | |  | Control group  n=64 | |
| --- | --- | --- | --- | --- | --- |
| Age (yr) | 34.9±6.2 | |  | 36.1±5.5 | |
| Sex, Male (%) | 7 (20.0) | |  | 116 (31.9) | |
| BMI (kg/m^2^) | 23.5±4.3 | |  | 24.0±3.7 | |
| TSH (mIU/L) | 0.14 (0.06, 0.30) | |  | 0.10 (0.03, 0.31) | |
|  | Baseline | 12 months |  | Baseline | 12 months |
| 25OHD (ng/ml) | 13.9±3.9 | 31.8±9.7***^,^ **^###^** |  | 14.2±4.8 | 18.7±8.3** |
| PTH (pg/ml) | 46.6±16.5 | 35.3±11.9**^,^ **^#^** |  | 43.3±17.6 | 45.7±17.1* |
| ALP (U/L) | 68±17 | 63±14 |  | 69±20 | 70±23 |
| β-CTX (ng/ml) | 0.38±0.19 | 0.29±0.16*^,^ **^#^** |  | 0.41±0.18 | 0.39±0.22 |
| **TSH≥0.5 mIU/L** | Vit D group  n=47 | |  | Control group  n=100 | |
| Age (yr) | 35.5±5.8 | |  | 35.8±5.6 | |
| Sex, Male (%) | 12 (25.5) | |  | 22 (22.0) | |
| BMI (kg/m^2^) | 23.7±3.2 | |  | 23.5±3.5 | |
| TSH (mIU/L) | 0.14 (0.06, 0.30) | |  | 1.46 (0.84, 2.16) | |
|  | Baseline | 12 months |  | Baseline | 12 months |
| 25OHD (ng/ml) | 13.8±3.9 | 29.8±5.3***^,^ **^###^** |  | 14.3±4.3 | 16.9±5.1*** |
| PTH (pg/ml) | 44.6±19.3 | 36.9±13.4**^,^ **^##^** |  | 46.6±16.7 | 45.4±13.2 |
| ALP (U/L) | 66±17 | 59±16*** |  | 67±20 | 64±18 |
| β-CTX (ng/ml) | 0.36±0.19 | 0.26±0.33***^,^ **^#^** |  | 0.36±0.17 | 0.33±0.15 |

BMI: body mass index; TSH: thyroid stimulating hormone; 25OHD: 25-hydroxyvitamin D; PTH: parathyroid hormone; ALP: alkaline phosphatase, β-CTX: β-isomerized carboxy-telopeptide of type I collagen. Values were given as mean ± SD, number (proportion) or median (interquartile range). **P*<0.05, ***P*<0.01, ****P*<0.001 indicated significant difference compared with baseline. #*P*<0.05, ##*P*<0.01, ###*P*<0.001 indicated significant difference between treatment and control groups.

**Supplemental Table 2** Characteristics and outcomes of the unadjusted cohorts of DTC

|  | Vit D group  n=94 | |  | Control group  n=364 | |
| --- | --- | --- | --- | --- | --- |
| Age (yr) | 35.3±5.8 | |  | 36.1±5.5 | |
| Sex, Male (%) | 22 (23.4) | |  | 116 (31.9) | |
| BMI (kg/m^2^) | 23.4±3.7 | |  | 24.0±3.7 | |
| TSH (mIU/L) | 0.62 (0.15, 1.57) | |  | 0.66 (0.18, 1.59) | |
|  | Baseline | 12 months |  | Baseline | 12 months |
| 25OHD (ng/ml) | 13.7±3.8**^###^** | 29.6±7.8***^,^ **^###^** |  | 16.8±5.4 | 19.0±6.2** |
| PTH (pg/ml) | 46.7±18.6**^##^** | 37.2±13.0**^,^ **^##^** |  | 41.5±15.5 | 43.3±15.4* |
| ALP (U/L) | 68±20 | 61±16**^#^** |  | 67±17 | 66±19 |
| β-CTX (ng/ml) | 0.37±0.19 | 0.27±0.15*^,^ **^##^** |  | 0.36±0.17 | 0.34±0.16 |
| LS BMD (g/cm^2^) | 1.223±0.119**^##^** | 1.236±0.123* |  | 1.273±0.136 | 1.276±0.140* |
| FN BMD (g/cm^2^) | 0.945±0.110**^#^** | 0.943±0.115 |  | 0.979±0.120 | 0.972±0.116 |
| TROCH BMD (g/cm^2^) | 0.747±0.110**^##^** | 0.746±0.113*^,^ **^#^** |  | 0.787±0.108 | 0.785±0.114 |
| TH BMD (g/cm^2^) | 0.988±0.118**^#^** | 0.985±0.116* |  | 1.023±0.117 | 1.020±0.117 |

BMI: body mass index; TSH: thyroid stimulating hormone; 25OHD: 25-hydroxyvitamin D; PTH: parathyroid hormone; ALP: alkaline phosphatase, β-CTX: β-isomerized carboxy-telopeptide of type I collagen. Values were given as mean ± SD, number (proportion) or median (interquartile range). *: *P*<0.05, **: *P*<0.01, ***: *P*<0.001 indicated significant difference compared with baseline. #: *P*<0.05, ##: *P*<0.01, ###: *P*<0.001 indicated significant difference between treatment and control groups.
